# Supplementary material for: End Sequence Analysis Toolkit (ESAT) expands the extractable information from single-cell RNA-seq data
Source: Genome Res. 2016 Oct;26(10):1397–410. doi: 10.1101/gr.207902.116 (PMC5052061; doi:10.1101/gr.207902.116)
Supplement: Supplemental Material [file supp_gr.207902.116_Supp_Materials.tar.gz › ESAT-esat_v0.1/doc/html/classbroad_1_1pda_1_1seq_1_1protection_1_1_multi_sample_scan_peak_caller.html]

ESAT: broad.pda.seq.protection.MultiSampleScanPeakCaller Class Reference


ESAT


- Main Page
- Related Pages
- Packages
- Classes
- Files

- Class List
- Class Index
- Class Hierarchy
- Class Members

All Classes Namespaces Files Functions Variables Enumerator Pages

- broad
- pda
- seq
- protection
- MultiSampleScanPeakCaller

Classes |
Public Member Functions |
Static Public Member Functions |
Protected Member Functions |
Static Protected Member Functions |
Protected Attributes |
Static Protected Attributes |
List of all members

broad.pda.seq.protection.MultiSampleScanPeakCaller Class Reference

Inheritance diagram for broad.pda.seq.protection.MultiSampleScanPeakCaller:

[legend]

Collaboration diagram for broad.pda.seq.protection.MultiSampleScanPeakCaller:

[legend]

|  |  |
| --- | --- |
| Public Member Functions | |
|  | MultiSampleScanPeakCaller (String sampleListFile, String bedFile, String chrSizeFile, int window, int step) throws IOException |
|  | |
| void | setFilterByStrand (boolean useStrandFilter) |
|  | |
| void | setExtraFields (boolean useExtraFields) |
|  | |
| void | setBinomialScore (boolean binomialScore) |
|  | |
| void | setPeakCutoffMostCommonReplicate (double maxPct) |
|  | |
| void | setPeakTrimQuantile (double trimPeakQuantile) |
|  | |
| void | setPeakWindowCountCutoff (double peakCountCutoff) |
|  | |
| void | setPeakWindowScanPvalCutoff (double peakScanPvalCutoff) |
|  | |
| void | setFirstReadTranscriptionStrand (boolean firstReadIsTranscriptionStrand) |
|  | |
| void | setExpressionScanPvalueCutoff (double expressionScanPvalCutoff) |
|  | |
|  | MultiSampleScanPeakCaller (String expressionBamFile, String signalBamFile, String bedFile, String chrSizeFile) throws IOException |
|  | |
|  | MultiSampleScanPeakCaller (String expressionBamFile, String signalBamFile, String bedFile, String chrSizeFile, int window, int step) throws IOException |
|  | |
| boolean | isExpressedInAllControlSamples (Gene gene) |
|  | |
| boolean | isExpressed (Gene gene) |
|  | |
| Collection< Gene > | getSingleSampleScanPeaks (SampleData sample, Gene gene) throws IOException |
|  | |
| Collection< Annotation > | mergePeaks (Collection< Annotation > peaks) |
|  | |
| void | setCoordinateSpace (CoordinateSpace space) |
|  | |
| CoordinateSpace | getCoordinateSpace () |
|  | |

|  |  |
| --- | --- |
| Static Public Member Functions | |
| static void | main (String[] args) throws IOException, InterruptedException, DrmaaException |
|  | |

|  |  |
| --- | --- |
| Protected Member Functions | |
|  | MultiSampleScanPeakCaller (MultiSampleScanPeakCaller other) throws IOException |
|  | |
| void | initializeFilterRejectWriters (String commonSuffix, String outDir) throws IOException |
|  | |
| void | closeFilterRejectWriters () throws IOException |
|  | |
| String | getPeakBedFileName (SampleData sample, String outDir, String chrName) |
|  | |
| void | writeSingleSampleScanPeaks (SampleData sample, String outFile, String chrName) throws IOException |
|  | |
| SamplePermutation | getOneRandomSamplePermutation () |
|  | |
| void | setLoggerLevel (Level level) |
|  | |

|  |  |
| --- | --- |
| Static Protected Member Functions | |
| static MultiSampleScanPeakCaller | createFromCommandArgs (String[] commandArgs) throws IOException |
|  | |
| static boolean | commandLineHasDebugFlag (String[] commandArgs) |
|  | |
| static String | commandLineOutDir (String[] commandArgs) |
|  | |

|  |  |
| --- | --- |
| Protected Attributes | |
| Map< String, Collection< Gene > > | genes |
|  | |
| Map< SampleData, Map< Gene,   Map< Annotation,   BinomialEnrichmentScore > > > | binomialWindowScores |
|  | |
| GenomeSpaceSampleData | expressionData |
|  | |
| ArrayList< SampleData > | controlSamples |
|  | |
| ArrayList< SampleData > | signalSamples |
|  | |
| ArrayList< SampleData > | allSamples |
|  | |
| int | windowSize |
|  | |
| int | stepSize |
|  | |
| int | numControls |
|  | |
| int | numSignals |
|  | |
| int | numSamples |
|  | |
| Random | random |
|  | |
| String | sizeFile |
|  | |

|  |  |
| --- | --- |
| Static Protected Attributes | |
| static Logger | logger = Logger.getLogger(MultiSampleScanPeakCaller.class.getName()) |
|  | |
| static String | FILTER\_REJECT\_DIR = "filter\_rejects" |
|  | |

## Detailed Description

Author
:   prussell

## Constructor & Destructor Documentation

|  |  |  |  |  |  |
| --- | --- | --- | --- | --- | --- |
| broad.pda.seq.protection.MultiSampleScanPeakCaller.MultiSampleScanPeakCaller | ( | MultiSampleScanPeakCaller | *other* | ) | throws IOException |

protected

Here is the caller graph for this function:

|  |  |  |  |
| --- | --- | --- | --- |
| broad.pda.seq.protection.MultiSampleScanPeakCaller.MultiSampleScanPeakCaller | ( | String | *sampleListFile*, |
|  |  | String | *bedFile*, |
|  |  | String | *chrSizeFile*, |
|  |  | int | *window*, |
|  |  | int | *step* |
|  | ) |  | throws IOException |

Instantiate using file of sample information

Parameters
:   |  |  |
    | --- | --- |
    | sampleListFile | File containing sample list |
    | bedFile | Bed gene annotation |
    | chrSizeFile | Chromosome size file |
    | window | Window size |
    | step | Step size |

Exceptions
:   |  |  |
    | --- | --- |
    | IOException |  |

|  |  |  |  |
| --- | --- | --- | --- |
| broad.pda.seq.protection.MultiSampleScanPeakCaller.MultiSampleScanPeakCaller | ( | String | *expressionBamFile*, |
|  |  | String | *signalBamFile*, |
|  |  | String | *bedFile*, |
|  |  | String | *chrSizeFile* |
|  | ) |  | throws IOException |

Instantiate with a single sample and an expression sample and default paramters

Parameters
:   |  |  |
    | --- | --- |
    | expressionBamFile | Bam file for expression sample |
    | signalBamFile | Bam file for signal sample |
    | bedFile | Bed gene annotation |
    | chrSizeFile | Chromosome size file |

Exceptions
:   |  |  |
    | --- | --- |
    | IOException |  |

|  |  |  |  |
| --- | --- | --- | --- |
| broad.pda.seq.protection.MultiSampleScanPeakCaller.MultiSampleScanPeakCaller | ( | String | *expressionBamFile*, |
|  |  | String | *signalBamFile*, |
|  |  | String | *bedFile*, |
|  |  | String | *chrSizeFile*, |
|  |  | int | *window*, |
|  |  | int | *step* |
|  | ) |  | throws IOException |

Instantiate with a single signal sample and an expression sample

Parameters
:   |  |  |
    | --- | --- |
    | expressionBamFile | Bam file for expression sample |
    | signalBamFile | Bam file for signal sample |
    | bedFile | Bed gene annotation |
    | chrSizeFile | Chromosome size file |
    | window | Window size |
    | step | Step size |

Exceptions
:   |  |  |
    | --- | --- |
    | IOException |  |

## Member Function Documentation

|  |  |  |  |  |
| --- | --- | --- | --- | --- |
| void broad.pda.seq.protection.MultiSampleScanPeakCaller.closeFilterRejectWriters | ( |  | ) | throws IOException |

protected

|  |  |  |  |  |  |
| --- | --- | --- | --- | --- | --- |
| static boolean broad.pda.seq.protection.MultiSampleScanPeakCaller.commandLineHasDebugFlag | ( | String[] | *commandArgs* | ) |  |

staticprotected

Here is the caller graph for this function:

|  |  |  |  |  |  |
| --- | --- | --- | --- | --- | --- |
| static String broad.pda.seq.protection.MultiSampleScanPeakCaller.commandLineOutDir | ( | String[] | *commandArgs* | ) |  |

staticprotected

Here is the caller graph for this function:

|  |  |  |  |  |  |
| --- | --- | --- | --- | --- | --- |
| static MultiSampleScanPeakCaller broad.pda.seq.protection.MultiSampleScanPeakCaller.createFromCommandArgs | ( | String[] | *commandArgs* | ) | throws IOException |

staticprotected

Here is the call graph for this function:

Here is the caller graph for this function:

|  |  |  |  |  |
| --- | --- | --- | --- | --- |
| CoordinateSpace broad.pda.seq.protection.MultiSampleScanPeakCaller.getCoordinateSpace | ( |  | ) |  |

|  |  |  |  |  |
| --- | --- | --- | --- | --- |
| SamplePermutation broad.pda.seq.protection.MultiSampleScanPeakCaller.getOneRandomSamplePermutation | ( |  | ) |  |

protected

Get a random permutation of control and signal identities

Returns
:   The random permutation

|  |  |  |  |
| --- | --- | --- | --- |
| String broad.pda.seq.protection.MultiSampleScanPeakCaller.getPeakBedFileName | ( | SampleData | *sample*, |
|  |  | String | *outDir*, |
|  |  | String | *chrName* |
|  | ) |  |  |

protected

Get name of bed file to write for peaks

Parameters
:   |  |  |
    | --- | --- |
    | sample | Sample |
    | outDir | Output directory name or null if current directory |
    | chrName | Chromosome name or null if all chromosomes |

Returns
:   File name

|  |  |  |  |
| --- | --- | --- | --- |
| Collection<Gene> broad.pda.seq.protection.MultiSampleScanPeakCaller.getSingleSampleScanPeaks | ( | SampleData | *sample*, |
|  |  | Gene | *gene* |
|  | ) |  | throws IOException |

Get scan peaks for the sample and the gene

Parameters
:   |  |  |
    | --- | --- |
    | sample | The sample |
    | gene | The gene |

Returns
:   Significant scan peaks

Exceptions
:   |  |  |
    | --- | --- |
    | IOException |  |

Here is the caller graph for this function:

|  |  |  |  |
| --- | --- | --- | --- |
| void broad.pda.seq.protection.MultiSampleScanPeakCaller.initializeFilterRejectWriters | ( | String | *commonSuffix*, |
|  |  | String | *outDir* |
|  | ) |  | throws IOException |

protected

|  |  |  |  |  |  |
| --- | --- | --- | --- | --- | --- |
| boolean broad.pda.seq.protection.MultiSampleScanPeakCaller.isExpressed | ( | Gene | *gene* | ) |  |

Whether the gene is significantly expressed in the special expression sample

Parameters
:   |  |  |
    | --- | --- |
    | gene | The gene |

Returns
:   True iff the gene is expressed in the expression sample

|  |  |  |  |  |  |
| --- | --- | --- | --- | --- | --- |
| boolean broad.pda.seq.protection.MultiSampleScanPeakCaller.isExpressedInAllControlSamples | ( | Gene | *gene* | ) |  |

Whether the gene is expressed in all control samples at the given significance level

Parameters
:   |  |  |
    | --- | --- |
    | gene | The gene |

Returns
:   Whether the gene is expressed by these criteria

|  |  |  |  |  |  |
| --- | --- | --- | --- | --- | --- |
| static void broad.pda.seq.protection.MultiSampleScanPeakCaller.main | ( | String[] | *args* | ) | throws IOException, InterruptedException, DrmaaException |

static

Parameters
:   |  |  |
    | --- | --- |
    | args |  |

Exceptions
:   |  |  |
    | --- | --- |
    | IOException |  |
    | InterruptedException |  |
    | DrmaaException |  |

Here is the call graph for this function:

|  |  |  |  |  |  |
| --- | --- | --- | --- | --- | --- |
| Collection<Annotation> broad.pda.seq.protection.MultiSampleScanPeakCaller.mergePeaks | ( | Collection< Annotation > | *peaks* | ) |  |

|  |  |  |  |  |  |
| --- | --- | --- | --- | --- | --- |
| void broad.pda.seq.protection.MultiSampleScanPeakCaller.setBinomialScore | ( | boolean | *binomialScore* | ) |  |

Set whether to print extra fields

Parameters
:   |  |  |
    | --- | --- |
    | useExtraFields |  |

|  |  |  |  |  |  |
| --- | --- | --- | --- | --- | --- |
| void broad.pda.seq.protection.MultiSampleScanPeakCaller.setCoordinateSpace | ( | CoordinateSpace | *space* | ) |  |

|  |  |  |  |  |  |
| --- | --- | --- | --- | --- | --- |
| void broad.pda.seq.protection.MultiSampleScanPeakCaller.setExpressionScanPvalueCutoff | ( | double | *expressionScanPvalCutoff* | ) |  |

Set genome wide scan P value cutoff for expression of transcript

Parameters
:   |  |  |
    | --- | --- |
    | expressionScanPvalCutoff | P value cutoff for transcript expression against genomic background |

|  |  |  |  |  |  |
| --- | --- | --- | --- | --- | --- |
| void broad.pda.seq.protection.MultiSampleScanPeakCaller.setExtraFields | ( | boolean | *useExtraFields* | ) |  |

Set whether to print extra fields

Parameters
:   |  |  |
    | --- | --- |
    | useExtraFields |  |

|  |  |  |  |  |  |
| --- | --- | --- | --- | --- | --- |
| void broad.pda.seq.protection.MultiSampleScanPeakCaller.setFilterByStrand | ( | boolean | *useStrandFilter* | ) |  |

Set whether to use strand information in reads to filter peaks

Parameters
:   |  |  |
    | --- | --- |
    | useStrandFilter | Whether to apply strand filter |

|  |  |  |  |  |  |
| --- | --- | --- | --- | --- | --- |
| void broad.pda.seq.protection.MultiSampleScanPeakCaller.setFirstReadTranscriptionStrand | ( | boolean | *firstReadIsTranscriptionStrand* | ) |  |

Set whether read 1 is transcription strand

Parameters
:   |  |  |
    | --- | --- |
    | firstReadIsTranscriptionStrand | True if read 1 is transcription strand |

|  |  |  |  |  |  |
| --- | --- | --- | --- | --- | --- |
| void broad.pda.seq.protection.MultiSampleScanPeakCaller.setLoggerLevel | ( | Level | *level* | ) |  |

protected

Set logger levels for all samples

Parameters
:   |  |  |
    | --- | --- |
    | level | Level |

|  |  |  |  |  |  |
| --- | --- | --- | --- | --- | --- |
| void broad.pda.seq.protection.MultiSampleScanPeakCaller.setPeakCutoffMostCommonReplicate | ( | double | *maxPct* | ) |  |

Set cutoff for the percentage of fragments overlapping a peak that come from the most common replicate fragment

Parameters
:   |  |  |
    | --- | --- |
    | maxPct | The max percentage |

|  |  |  |  |  |  |
| --- | --- | --- | --- | --- | --- |
| void broad.pda.seq.protection.MultiSampleScanPeakCaller.setPeakTrimQuantile | ( | double | *trimPeakQuantile* | ) |  |

Set quantile for trim max contiguous algorithm for peak calling

Parameters
:   |  |  |
    | --- | --- |
    | trimPeakQuantile | The quantile of data values |

|  |  |  |  |  |  |
| --- | --- | --- | --- | --- | --- |
| void broad.pda.seq.protection.MultiSampleScanPeakCaller.setPeakWindowCountCutoff | ( | double | *peakCountCutoff* | ) |  |

Set minimum number of fragments overlapping a window to be considered for possible peak

Parameters
:   |  |  |
    | --- | --- |
    | peakCountCutoff | Minimum fragment count for each window |

|  |  |  |  |  |  |
| --- | --- | --- | --- | --- | --- |
| void broad.pda.seq.protection.MultiSampleScanPeakCaller.setPeakWindowScanPvalCutoff | ( | double | *peakScanPvalCutoff* | ) |  |

Set scan P value cutoff for window within transcript to be considered for possible peak

Parameters
:   |  |  |
    | --- | --- |
    | peakScanPvalCutoff | Max scan P value |

|  |  |  |  |
| --- | --- | --- | --- |
| void broad.pda.seq.protection.MultiSampleScanPeakCaller.writeSingleSampleScanPeaks | ( | SampleData | *sample*, |
|  |  | String | *outFile*, |
|  |  | String | *chrName* |
|  | ) |  | throws IOException |

protected

Write all single sample scan peaks for the sample and chromosome to file

Parameters
:   |  |  |
    | --- | --- |
    | sample | The sample |
    | outFile | Output bed file |
    | r | Red value for bed file color |
    | g | Green value for bed file color |
    | b | Blue value for bed file color |
    | chrName | Only write peaks for this chromosome |

Exceptions
:   |  |  |
    | --- | --- |
    | IOException |  |

Here is the call graph for this function:

## Member Data Documentation

ArrayList<SampleData> broad.pda.seq.protection.MultiSampleScanPeakCaller.allSamples

protected

Map<SampleData, Map<Gene, Map<Annotation, BinomialEnrichmentScore> > > broad.pda.seq.protection.MultiSampleScanPeakCaller.binomialWindowScores

protected

ArrayList<SampleData> broad.pda.seq.protection.MultiSampleScanPeakCaller.controlSamples

protected

GenomeSpaceSampleData broad.pda.seq.protection.MultiSampleScanPeakCaller.expressionData

protected

String broad.pda.seq.protection.MultiSampleScanPeakCaller.FILTER\_REJECT\_DIR = "filter\_rejects"

staticprotected

Map<String, Collection<Gene> > broad.pda.seq.protection.MultiSampleScanPeakCaller.genes

protected

Logger broad.pda.seq.protection.MultiSampleScanPeakCaller.logger = Logger.getLogger(MultiSampleScanPeakCaller.class.getName())

staticprotected

int broad.pda.seq.protection.MultiSampleScanPeakCaller.numControls

protected

int broad.pda.seq.protection.MultiSampleScanPeakCaller.numSamples

protected

int broad.pda.seq.protection.MultiSampleScanPeakCaller.numSignals

protected

Random broad.pda.seq.protection.MultiSampleScanPeakCaller.random

protected

ArrayList<SampleData> broad.pda.seq.protection.MultiSampleScanPeakCaller.signalSamples

protected

String broad.pda.seq.protection.MultiSampleScanPeakCaller.sizeFile

protected

int broad.pda.seq.protection.MultiSampleScanPeakCaller.stepSize

protected

int broad.pda.seq.protection.MultiSampleScanPeakCaller.windowSize

protected

---

The documentation for this class was generated from the following file:

- java/broad/pda/seq/protection/MultiSampleScanPeakCaller.java


---

Generated on Fri Aug 15 2014 15:11:03 for ESAT by  

 1.8.7
